# Media Reporting of Neuroscience Depends on Timing, Topic and Newspaper Type

**DOI:** 10.1371/journal.pone.0104780

**Published:** 2014-08-12

**Authors:** Nienke M. van Atteveldt, Sandra I. van Aalderen-Smeets, Carina Jacobi, Nel Ruigrok

**Affiliations:** 1 Department of Educational Neuroscience, Faculty of Psychology and Education and Institute Learn!, VU University Amsterdam, Amsterdam, The Netherlands; 2 Department of Cognitive Neuroscience, Faculty of Psychology & Neuroscience, Maastricht University, Maastricht, The Netherlands; 3 Centre for Science Education and Talent Development (SETD), Faculty of Behavioural Sciences, University of Twente, Enschede, The Netherlands; 4 Department of Media & Communication, Erasmus University, Rotterdam, The Netherlands; 5 Department of Communication, University of Vienna, Vienna, Austria; University of Missouri-Kansas City, United States of America

## Abstract

The rapid developments in neuroscientific techniques raise high expectations among the general public and therefore warrant close monitoring of the translation to the media and daily-life applications. The need of empirical research into neuroscience communication is emphasized by its susceptibility to evoke misconceptions and polarized beliefs. As the mass media are the main sources of information about (neuro-)science for a majority of the general public, the objective of the current research is to quantify how critically and accurately newspapers report on neuroscience as a function of the timing of publication (within or outside of periods of heightened media attention to neuroscience, termed “news waves”), the topic of the research (e.g. development, health, law) and the newspaper type (quality, popular, free newspapers). The results show that articles published during neuroscience news waves were less neutral and more optimistic, but not different in accuracy. Furthermore, the overall tone and accuracy of articles depended on the topic; for example, articles on development often had an optimistic tone whereas articles on law were often skeptical or balanced, and articles on health care had highest accuracy. Average accuracy was rather low, but articles in quality newspapers were relatively more accurate than in popular and free newspapers. Our results provide specific recommendations for researchers and science communicators, to improve the translation of neuroscience findings through the media: 1) Caution is warranted during periods of heightened attention (news waves), as reporting tends to be more optimistic; 2) Caution is also warranted not to follow topic-related biases in optimism (e.g., development) or skepticism (e.g., law); 3) Researchers should keep in mind that overall accuracy of reporting is low, and especially articles in popular and free newspapers provide a minimal amount of details. This indicates that researchers themselves may need to be more active in preventing misconceptions to arise.

## Introduction

Modern neuroscience research, including neuro-imaging techniques such as functional magnetic resonance imaging (fMRI), enables exploring the living human brain with unprecedented accuracy. Not surprisingly, these recent developments in neuro-imaging raise high expectations in society, which is illustrated by the proliferation of “brain-based” teaching methods in education [1],[2], the emergence of biomarkers for psychiatric illnesses [3], or recent debates on neuroscience and the law [4], [5]. These high societal expectations are also reflected by large-scale funding schemes such as the recent US-based BRAIN initiative or the European Human Brain Project [6]. At the same time, neuro-imaging advances have also received skepticism [7], [8] and actual applicability has been very limited [3], [9], [10]. This is reminiscent of the promise-disappointment cycles identified in societal expectations of biotechnology [11] and indicates that the public image of neuroscience may not be realistic, but is often positively or negatively biased. The translation of neuro-imaging research to the public and daily life applications is not straightforward and sensitive to misconceptions ([12], [13], [14]; but see [15]). For example, common myths are that we only use 10% of our brain [16], or the idea that children are either “left-brained” or “right-brained” learners [2], [12]. The media are thought to be an important factor in reinforcing such misconceptions as important details are often omitted in press articles [17]. Moreover, many applications of neuro-imaging research are ethically sensitive, for example when findings are associated with stigmatization of certain groups [18]. The susceptibility to misconceptions and the ethically complex nature of many applications highlight the importance of accurate transmission of neuroscientific results. Therefore, more empirical research into this communication process is needed [13].

Prior to media reporting, the translation of brain imaging research to daily life applications and mainstream “knowledge” includes many steps that all have their own challenges. Dissemination of brain research findings to the general public by (print) media is one of the final stages in the translation process, and a very important one, as the mass media are the main sources of information about science in general, and neuroscience specifically [19], for a majority of the general public [20], [21]. Although the current work only covers the translation step of media reporting, we will begin with a quick overview of the translation steps preceding media reporting, and how they are sensitive to misconceptions. Generally, the chain of steps include: 1) the noise and uncertainties of the measurement technique (e.g. [22]) of which the general public may not be aware; 2) analysis and selection of results, which depends on the choices the researcher makes such as analytical approach [23]; 3) interpretation and framing of results for publication in a scientific journal. This step typically includes optimism about, or even overstating [24], [25] the benefits and applicability in the conclusions which might lead to inflated expectations by the public [10]; and 4) the issuing of press releases by communication departments who tend to take over the inflated optimism [26], and whose quality strongly influences the quality of associated newspaper coverage [27]. Dissemination of research findings to the general public by the mass media [28], [29], [30] is the final step and the focus of this research. It should be noted that neuroscience results can also enter the practice more directly, for example through experts or consultants in clinics, companies or governmental departments. These forms of translation are not covered by the present work.

Given this complex translation process, it may be unavoidable that research results are to some extent simplified and generalized when they appear in the media. The current challenge is to guard correct transfer of research methods and results, and realism regarding the interpretation and applicability, to such an extent that unjustified expectations (or fear) and misconceptions will be avoided [17]. To achieve this, it is important that media coverage of neuroscience is both accurate and critical. In regards to *accuracy*, it is important that enough details about the research are included in the article [30], [31]. The subset of important details used in the current study are whether or not the research technique is specified, whether this technique is explained, whether or not the tested species is mentioned to avoid animal-to-human generalization, and whether or not the scientific journal in which the study was published is mentioned. The more of these details are included, the less likely it is that the original research findings reach the public in a distorted way. Additional important details may differ across specific research technique or topic, such as details on the experimental design and resemblance to real-life processes, but these are not covered by the current broad analysis. For being *critical*, it is important that risks, challenges and/or limitations of the research (e.g. uncertainties in the technique, generalizability, lack of power, etc.) are considered side by side to benefits and possibilities for applications such as treatments [29], [30]. Overly optimistic reporting on neuroscience topics has been shown before [32] and has the risk of raising unrealistic expectations. The more balanced an article is in terms of discussing both benefits and challenges, the better the public will be able to form realistic beliefs and expectations about neuroscience and its potential applications.

How critical and accurate reporting on neuroscience is may depend on several factors. First of all, a critical view and sufficient accuracy may be compromised during periods of heightened media attention, which we call “news waves”. Media attention to certain topics, such as new findings or controversial statements about neuroscience, is often concentrated in time during such news waves. It has been shown that especially during news waves, journalistic principles, such as checking information and presenting both sides of a story, may be compromised [33], [34] and media tend to follow each other in what and how they report. This may result in lower accuracy and a less balanced tone in articles covering neuroscience during news waves. We use the term “news wave” to avoid confusion with the term “hype” that has been used in various ways in other research on media coverage of science. For example, previous research has used “hype” to indicate overstated conclusions and unbalanced portrayal of benefits over limitations and potential risks in relation to genetic technologies [35] and neuro-enhancement techniques [32], without including any aspect of reporting dynamics. In communication science, the term media hype is typically used to indicate a certain media dynamic, a period of self-reinforcing heightened attention to a certain topic [34], which is the basis for how we defined “news waves”, i.e., periods in time in which significantly more articles on neuroscience are published than on average.

Secondly, how critical and accurate reporting is may depend on the topic. For example, Racine and colleagues [29] showed differences in critical view of articles reporting on health versus non-health related issues. Here, we extend the range of topics, motivated by certain predictions. For example, it has recently been shown that misconceptions about neuroscience are abundant among school teachers ([12]; but see [36]). The proliferation of such misconceptions (also termed “neuromyths”) is thought to be stimulated by the generally high motivation of teachers to apply knowledge about the brain [37], together with the growing availability of “brain-based” learning methods, which are often only very loosely based on neuroscientific evidence [1], [2]. This may suggest that transmission of neuroscience results in the context of development and learning may tend to be biased towards optimism. In contrast, a recent survey showed skepticism among the public about neuroimaging applications within law, safety and commercial domains, such as lie detection, employment screening or marketing research [19]. The same survey showed a positive attitude towards using neuroimaging for medical purposes, which is in line with the receptivity of patients and care providers to brain imaging found in other studies [38], [39].

Finally, reporting on neuroscience is expected to depend on the type of newspaper and the article type. Different newspapers have a different target audience and focus, and can be divided into quality, popular and free newspapers [40]. Hijmans and colleagues found that Dutch quality newspapers report more on science than popular newspapers [31]. The same study also found differences in tone (of general scientific reporting) between quality and popular newspapers, but no differences in accuracy. Specific to neuroscience reporting, Racine and colleagues did compare different sources of media, such as newspapers versus news magazines [29], but different types of newspapers have not been compared before. As free and popular newspapers have broader readership, it is important to relate the accuracy and critical view of reporting to how many people are reached by that information. For neuroscientists who engage with the media, it is important to have insight in how different newspapers typically represent neuroscience research. The Dutch print media system provides a good environment to address this issue, as different types of print newspapers exist that all have a good distribution, but differ considerably in readership [40]. In addition to newspaper type, also within newspapers, different article types have different communication goals [41]. For example, the goal of news articles is to inform readers about events, whereas commentaries fall in the category of “orienting journalism” and serve to facilitate interpreting events and developments. News articles are therefore predicted to have higher accuracy, and a less colored tone, than commentaries. Therefore, in addition to newspaper type, we predict that article type may also influence the characteristics of neuroscience reporting.

Several previous media-analyses have revealed important insights into how neuroscience research reaches the public [28], [29], [30]. The first study was focused on fMRI and showed a strong increase in media coverage since the early nineties, but the articles rarely were critical in tone and ethical issues were not well represented [29], [42]. In a next (larger-scale) study that included other scanning technologies as well, it was found that media articles contain only very limited details about the research [30]. Another recent study used a broader definition of brain research, but confined the content analysis to the subject of research. The study characterized the dominating themes in which neuroscience findings reach the public, such as the brain as index of difference among people, or as biological proof of traits or phenomena [28]. In sum, these previous studies focused on thematic representation of neuroscience in the media, and the forthcoming ethical, social and policy implications. Here, we keep the broad definition of brain research, but move beyond theme, overall tone and level of detail. Instead, we take a more in-depth approach and analyze how critical and accurate media coverage is as a function of timing (news wave), topic and newspaper type. We use a novel definition of ‘news wave’, to gain insight in whether or not neuroscience reporting is different during periods of heightened media attention. Moreover, the current work will assess the generalizability of previous findings that were mostly centered on UK media.

To summarize, our specific research aims are to characterize how *accurate* (or *detailed*) and *critical* newspaper reporting of neuroscience research is as a function of 1) timing (news wave or regular period), 2) topic of the research (development/learning, law/safety, politics/industry, philosophy/futuristic, health care), and 3) the type of newspaper (quality, popular and free newspapers) in which it was published. We predicted that reporting during news waves is less critical and less accurate than during regular periods, that reporting on topics related to brain development and learning/education is positively colored compared to other topics, and that critical view and accuracy are both lower in free and popular newspapers compared to quality newspapers. The results will enable us to provide neuroscientists, science communicators, and journalists with specific recommendations for improving the critical view and accuracy of neuroscience coverage by the media. The recommendations are specific in terms of focusing attention to the period of reporting (e.g., should one be extra careful during news waves?), the topic (e.g. should accuracy and critical view be guarded more strongly for certain topics compared to others?) and the newspaper type (e.g. should a scientist be extra alert in guarding correct communication to free/popular newspapers?).

## Materials and Methods

### Article selection and coding

We selected all articles reporting on neuroscientific research in 2008–2012 from six Dutch national daily newspapers that form a representative selection of quality (*de Volkskrant, NRC Handelsblad, Trouw*), popular (*De Telegraaf* and *Algemeen Dagblad*) (Hijmans et al., 2003), and free newspapers (*Spits* and *Metro*). Among the three quality newspapers included in the study, *De Volkskrant* is known as a progressive, left wing newspaper, *Trouw* was founded as an orthodox protestant newspaper representing the Christian part of the Netherlands, and *NRC Handelsblad* affiliated with a more liberal political viewpoint [43]. Among the two popular newspapers, *De Telegraaf* is the most widely read newspaper in the Netherlands and considered a populist right wing newspaper [44] and *Algemeen Dagblad* is the second largest subscription-based newspaper of the Netherlands with a strong focus on sports. The included free newspapers *Metro* and *Spits* are the most widely read free dailies in the Netherlands [40]. To select the relevant articles, we used the following search string:

((“brein onderzoek”∼10 OR “hersen* onderzoek”∼10 OR “neuro* onderzoek”∼10 OR hersenonderzoek OR hersenscan*) NOT (“brein achter” “stichting brein” “creatieve brein”))

This means we searched for the words “brain” (“brein”, “hersen*”) and “neuro” that were combined with “research” (“onderzoek”) within a distance of 10 words. We also searched for the words “brain scan” (“hersenscan”) and the Dutch compound word for brain research (“hersenonderzoek”). We excluded articles that were selected based on “brein achter” which means “the brain/mastermind behind”, “stichting Brein” (a Dutch anti-piracy foundation), and “creative brein” which means the creative mind behind something. We searched the entire articles, including headlines, lead paragraphs and body.

The selected articles (see table 1) have been coded by 3 experienced and independent coders using AmCAT (www.amcat.vu.nl), an online database and infrastructure for content analysis. The articles were coded on the article level using 14 coding questions (see table 2) with an extensive coding instruction (see Appendix S1). This instruction has been developed by the researchers on the basis of 4 test sets. These sets have been coded by the coders and the researchers themselves to eliminate ambiguities. The coding questions 1–6 (table 2) focused on assessing accuracy, critical view and topic of the articles. Article type (question 7) was used to gain more insight in the article type of articles showing experimental effects (post-hoc). Questions 8–14 were not relevant for the current research aims. The inter-coder reliability was assessed using 14 randomly selected articles, resulting in a sufficiently high Krippendorff's alpha of 0,78 [45].

**Table 1:** Details of the coded articles.

Newspaper Category	Newspaper	Number of selected articles 2008–2012	(%)	Average Print Run*	Average Readership**
**Quality Newspapers**	NRC Handelsblad	*310*	*28,7%*	*186,191*	*519,667*
	de Volkskrant	*309*	*28,6%*		
	Trouw	*146*	*13,5%*		
**Popular Newspapers**	Algemeen Dagblad	*119*	*11,0%*	*509,547*	*1,738,000*
	De Telegraaf	*109*	*10,1%*		
**Free Newspapers**	Metro	*44*	*4,1%*	*394,955*	*1,384,500*
	Spits	*38*	*3,5%*		
	**Total**	***1080***	***100%***		

^*^ Source: Institute for Media Auditing (HOI), http://www.hoi-online.nl/.

^**^Source: National Research Multimedia (NOM), http://www.nommedia.nl/.

**Table 2:** Coding questions.

*Q*	*Coding question*	*(Number of options) Answer options*
	**ACCURACY**	
1	On what technique does the article report?	(14) Functional MRI, MRI unspecified/“brain scan”, Anatomical, Electro-encephalography (EEG), Magneto-encephalography (MEG), Brain stimulation unspecified, Magnetic stimulation (TMS), Electrical stimulation (DBS, etc), Positron-emission tomography (PET), Optical techniques (NIRS, etc), Brain-computer-interface, Psychopharmacology, Other specified, Unspecified
2	Is the technique explained in the article?	(3) Yes - in 2 or more lines, Yes - in 1 line, No
3	What species was tested in the research?	(3) Humans, Animals, Not mentioned
4	Is the scientific journal in which the research is published mentioned?	(2) Yes, No
	**CRITICAL VIEW**	
5	What is the tone of the article?	(4) Optimistic, Skeptical, Neutral, Balanced
	**CONTENT**	
6	What is the main topic of the article?	(6) Development/Learning, Industry/Politics, Philosophy/Futuristic, Health care/Public health, Law/Safety, Other
	**OTHER**	
7	What is the article type?	(8) News report, Background, Person in the news, Editorial comment, Comment by newspaper columnist, External comment, Reader's letter, Service journalism (book reviews, etc.)
8	Are researchers of the reported work consulted as a source?	(2) Yes, No
9	Does the article report on the healthy brain or a brain disorder?	(5) Healthy-general, Healthy-development, Disorder-psychiatric, Disorder-neurological, Disorder- both psychiatric and neurological
10	What change or effect was found by the research?	(4) Improvement/increase, No change or effect, Worsening/decrease, No Effect intended
11	Does the article generalize from animal research to human implications? If yes, with or without explanation?	(4) Yes with explanation, Yes without explanation, No, Not applicable (in case the answer to q3 was “humans” or “not mentioned”)
12	Are independent experts or someone from the practice consulted as a source?	(2) Yes, No
13	What brain function was investigated?	(9) Memory, Motor functions, Attention, Sleep/Consciousness, Perception/Illusions, Social/Emotions, Cognition/language, Planning/Control/Free will, General/Multiple
14	What is the main message of the article?	(5) Emphasizing group differences, (new) application of brain research, Effect of a substance, Rhetoric (brain research used to support an argument), Other

### Analysis - dependent variables

The main research questions were how *accurate* and *critical* newspaper reporting of neuroscientific research is. The dependent variables “accuracy” and “critical view” were operationalized as follows:

#### Accuracy

Accuracy, defined as the level of detail, of an article was assessed by a combination of four coding questions (questions 1–4, see tables 2 & 3). Each article received a score of 0 or 1 for each of these 4 questions, and the total score for “ACCURATE” was the average of these 4 scores, resulting in a total score between 0 and 1. For example, an article that does not specify or explain the technique (2 * score  =  0) but does mention the tested species (e.g. human participants, score  =  1), and also mentions the journal where the work has been published (score  =  1), receives a total score for “ACCURATE” of (0+0+1+1)/4 = 0.5. We note that to be able to include all articles covering neuroscience, instead of focusing on specific techniques, the aspects included in this combined variable for “accuracy” are limited and technically focused. These limitations will be discussed in the Discussion.

**Table 3:** Coding questions used for calculating the value of the composite variable “ACCURATE”.

Coding question	Not Accurate (score = 0)	Accurate (score = 1)
Technique (q1)	*Unspecified; MRI unspecified/brain scan*	*Specific technique (fMRI, EEG, TMS etc.); Other specified*
Technique explained? (q2)	*No*	*Yes, in 1 line; Yes, in 2 or more lines*
Tested species (q3)	*Not mentioned*	*Animals; Humans*
Scientific journal as source? (q4)	*No*	*Yes*

#### Critical View

The coding question about the overall Tone of the article (question 5, table 2) was used to assess how critical the article is [29]. Tone has four categories: Balanced, Skeptical, Neutral and Optimistic. The category Balanced is considered as being a critical report of the research, as it includes both benefits and limitations. Articles in the categories Skeptical and Optimistic are negative vs. positive, as they only mention limitations or benefits, but not both. Neutral articles do not mention any benefits or limitations. It should be noted that in previous research, somewhat different definitions have been used for the different overall tone categories. For example, Racine and colleagues [27] used the category “critical” to indicate what we refer to as “skeptical”, and even thought the categories “balanced” are the same categories, we refer to “balanced” as being “most critical” as it includes both risks and benefits. The category “uncritical” used in their earlier study [26] would correspond to our categories “neutral” and “optimistic” combined. In contrast to the composite variable for Accuracy, we analyzed Tone separately as a categorical variable as we did not have complete a priori predictions for assigning values to the different Tone-categories (i.e., it is a truly nominal variable), except that we consider the Balanced category as most critical. Table 4 summarizes the variables used to assess critical view and accuracy of the articles.

**Table 4:** Summary and specification of the dependent variables.

Research question	Variable	Type	Values/Categories
How accurate?	ACCURATE	Continuous	Between 0 and 1
How critical?	TONE*	Categorical (nominal)	Optimistic, Skeptical, Neutral, Balanced

^*^ TONE was subsequently used as *independent* variable for the post-hoc analysis of the effect of tone on accuracy.

### Analysis - independent variables

We further specified the research question of how *accurate* and *critical* newspaper reporting of neuroscience research is, by asking whether the value/category of these two variables depends on the *timing* of publication, the *topic* of the article, and *newspaper type* of publication. Timing refers to whether or not an article is published during a period of heightened media attention to neuroscience: *news waves*. News waves were defined as periods of 6 consecutive days on which the number of articles about neuroscience research was 2 or more standard deviations above the average number per week (for that year). The newspapers in our analysis are not published on Sundays; therefore Sundays were excluded from this analysis. Topic was coded with the question about “topic” (question 6, table 2). For newspaper type, we used the categories explained in table 1: Quality, Popular and Free newspapers.

As indicated below table 4, we additionally used TONE as independent variable to assess the effect of TONE on ACCURACY, see below. For another post-hoc analysis we used Article Type as independent variable. Based on coding question 7 (table 2), we categorized the articles into News (News Report, Background and Person in the News), Commentaries (Editorial commentary, Commentary by newspaper columnist, External commentary, and Reader's letter) and Other (Service journalism). The independent variables are summarized in table 5.

**Table 5:** Summary and specification of the independent variables.

The effect of….	Variable	Type	Categories
Timing	NEWS WAVE	categorical	News wave, regular period
Topic	TOPIC	categorical	Industry/Politics, Philosophy, Health care, Law/Safety, Development/Learning, Other
Newspaper type	MEDIA TYPE	categorical	Quality, Popular, Free
Article type	ARTICLE TYPE	categorical	News, Commentaries, Other

### Statistical analysis

All statistical analyses were performed using SPSS (IBM, USA) and R (Revolution Analytics Headquarters, USA).

#### Planned analyses

With the dependent variable ACCURATE not distributed normally (W = 60690.5; p<.001) we decided to use a Mann-Whitney U Test to analyze effects of NEWS WAVE on the composite (continuous) dependent variable ACCURATE. To analyze effects of TOPIC and MEDIA TYPE we use Kruskal Wallis Tests with consequently all pairwise comparisons using Behrens Fisher Tests, while controlling the type I error rate (the probability of finding a difference that is not there) to assess which pairs were significantly different from each other. For the categorical, nominal dependent variable TONE, we tested for different distributions of the tone-categories by TOPIC, NEWS WAVE and MEDIA TYPE using Pearson's Chi-squared (X^2^) tests.

#### Post-hoc analyses

After inspecting the results of all planned analysis, we analyzed the effect of tone on accuracy, by running a Kruskal Wallis Test including all pairwise comparisons, on the ACCURATE values with TONE as independent variable. Moreover, we performed the same analysis including also MEDIA TYPE, resulting in a 2-factor (TONE, MEDIA TYPE) Kruskal Wallis Test, including all pairwise comparisons using Behrens Fisher Tests on the ACCURATE values. To gain insight in the type of articles in the different tone categories, we tested for different distributions of the tone-categories by ARTICLE TYPE using Pearson's Chi-squared (X^2^) tests. Finally, we tested differences in accuracy for the different article types using a Kruskal Wallis Test including all pairwise comparisons on the ACCURATE values with ARTICLE TYPE as independent variable.

## Results

### 1. General overview: how accurate and critical are newspaper articles on neuroscience research?

#### 1.1. Accuracy

**Figure 1:**
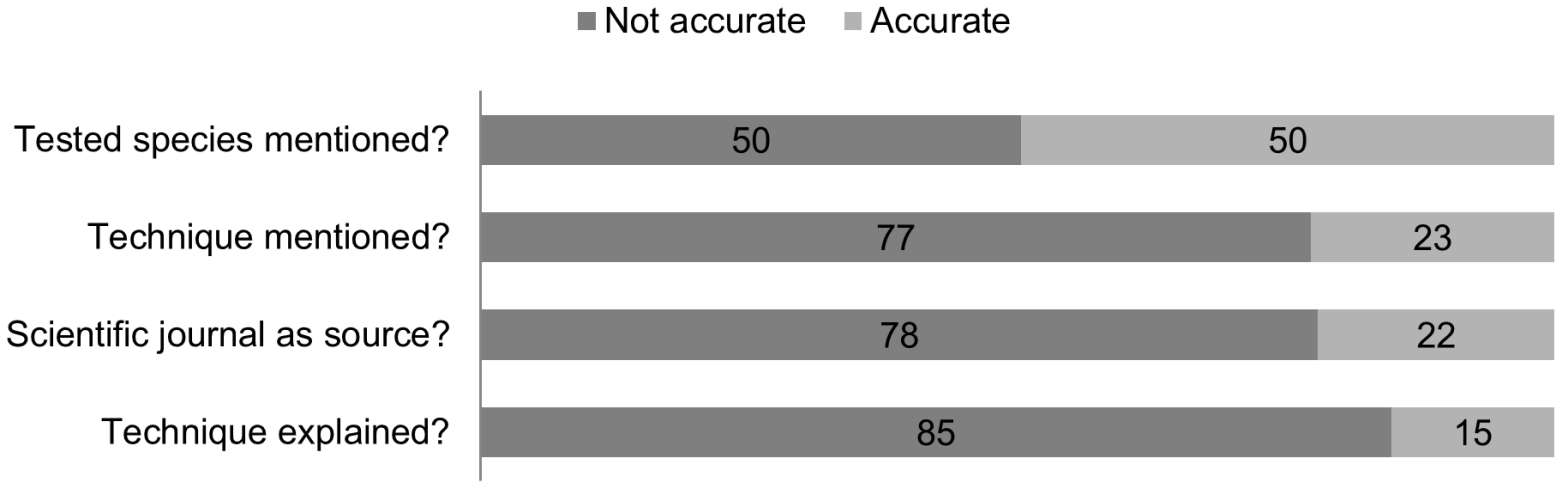
Proportions of “accurate” and “not accurate” scores per coding question included in the ACCURATE variable. The proportions (in %) of categorization into “not accurate” (value  =  0) versus “accurate” (value  =  1) for the 4 coding questions (see text left to the bar graph) that were used to calculate the value of the composite variable “ACCURATE”. Each article received a score of 0 or 1 for each of these 4 questions, and the total score for “ACCURATE” was the average of these 4 scores, resulting in a total score between 0 and 1.

In total, 1080 articles reported about neuroscience research in the period of 2008–2012 in the selected newspapers (table 1). Across all articles, the average value for the composite variable ACCURATE was 0.27 on a scale from 0 to 1. This means that from the 4 criteria we defined for accuracy (table 3), on average, only 1 was met. In figure 1, we show the proportions of categorization into “not accurate” (value  =  0) versus “accurate” (value  =  1) for these 4 criteria, to give insight in the origin of this relatively low score. It seems that especially few details are given on the technique of the reported research (only 23% of the articles mentioned the technique and only 15% explained the technique). Also, the scientific journal is cited only in about one 5^th^ of the articles. The tested species is mentioned in half of the articles.

#### 1.2 Critical view

Across all articles, the majority of 57% was neutral in tone, 13% had a balanced tone, 13% had a skeptical tone, and 17% were optimistic. This indicates that overall, neuroscience reporting is not very critical; only 13% of the articles discussed both benefits as well as limitations of the research (balanced). In the following, we will analyze how accuracy and critical view depends on the timing of publication (within news waves or not), the topic of the article and the type of newspaper in which an article was published (quality, popular or free newspaper).

### 2. TIMING: is reporting on neuroscience less accurate and critical during news waves than during regular periods?

*News waves* were defined as periods of 6 consecutive days on which the number of articles about neuroscience research was 2 or more standard deviations above the average number per week (for that year). In this definition, 22%–25% of all articles on neuroscience were reported during news waves (22% in quality newspapers, 24% in free newspapers, 25% in popular newspapers).

#### 2.1 Accuracy

The mean level of accuracy was lower during news waves (0.26) than during regular periods (0.28), but this difference was not significant (Mann-Whitney test of ACCURATE by NEWS WAVE: *U*(106985), p = 0.22.

#### 2.2 Critical view

**Figure 2:**
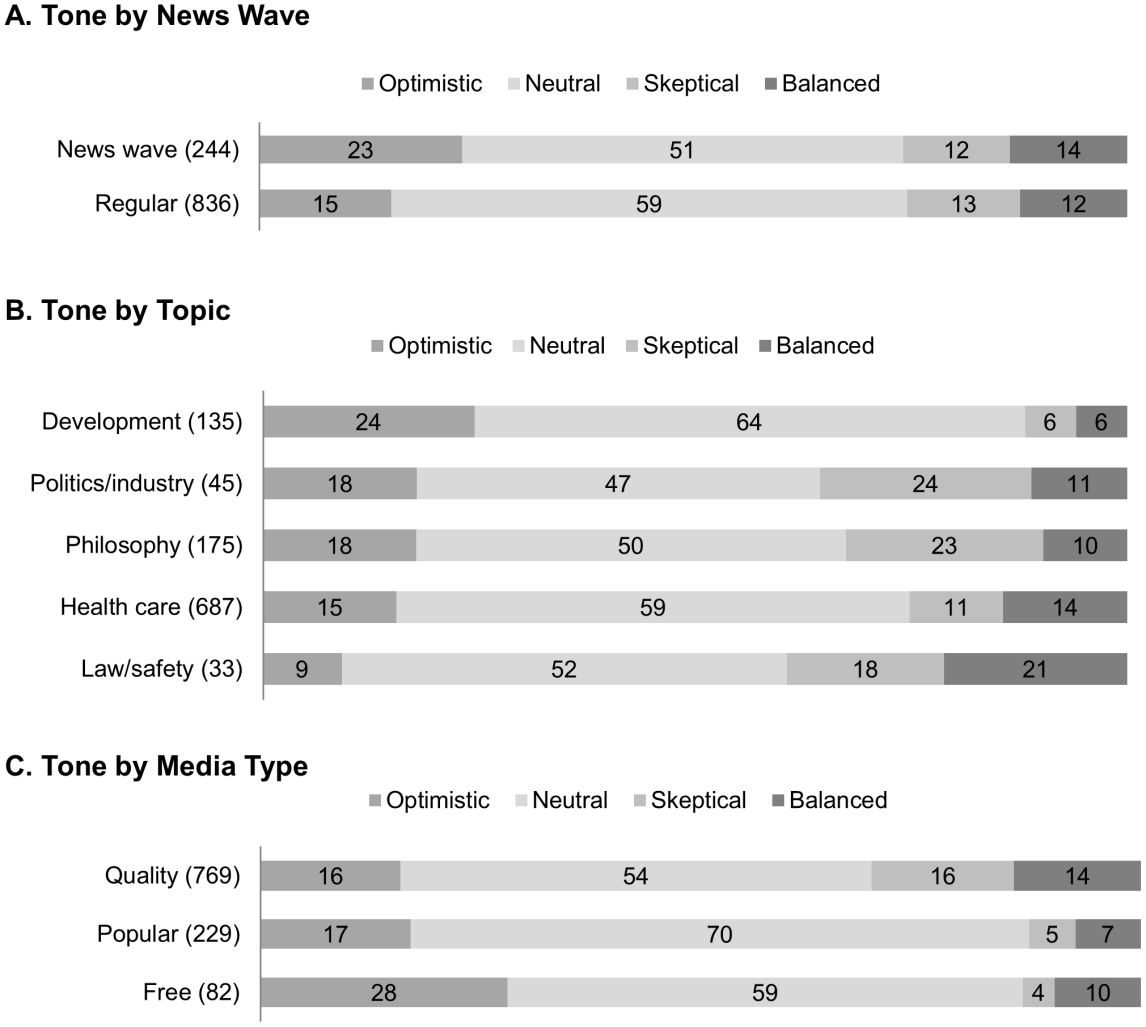
Distributions of Tone categories by News Wave, Topic, and Media Type. Values inside the bars are the percentages of the tone categories (see gray-scale coding legend above the bar graphs) within each category of the independent variables. Absolute numbers of articles are indicated between brackets behind the News wave, Topic and Media Type categories left to the bar graphs. A. Tone distribution for News wave versus regular reporting periods. B. Tone distribution for the different Topics. C. Tone distribution for the different Media Types.

Tone categories showed significantly different distributions during news waves (Figure 2A): TONE by NEWS WAVE, X^2^ (3) = 10.1, p<0.05. This difference was due to more optimistic and fewer neutral articles during news waves than during regular reporting periods. The proportion of balanced and skeptical articles was not different.

### 3. TOPIC: does accuracy and critical view of articles depend on the reported topic?

#### 3.1 Accuracy

**Figure 3:**
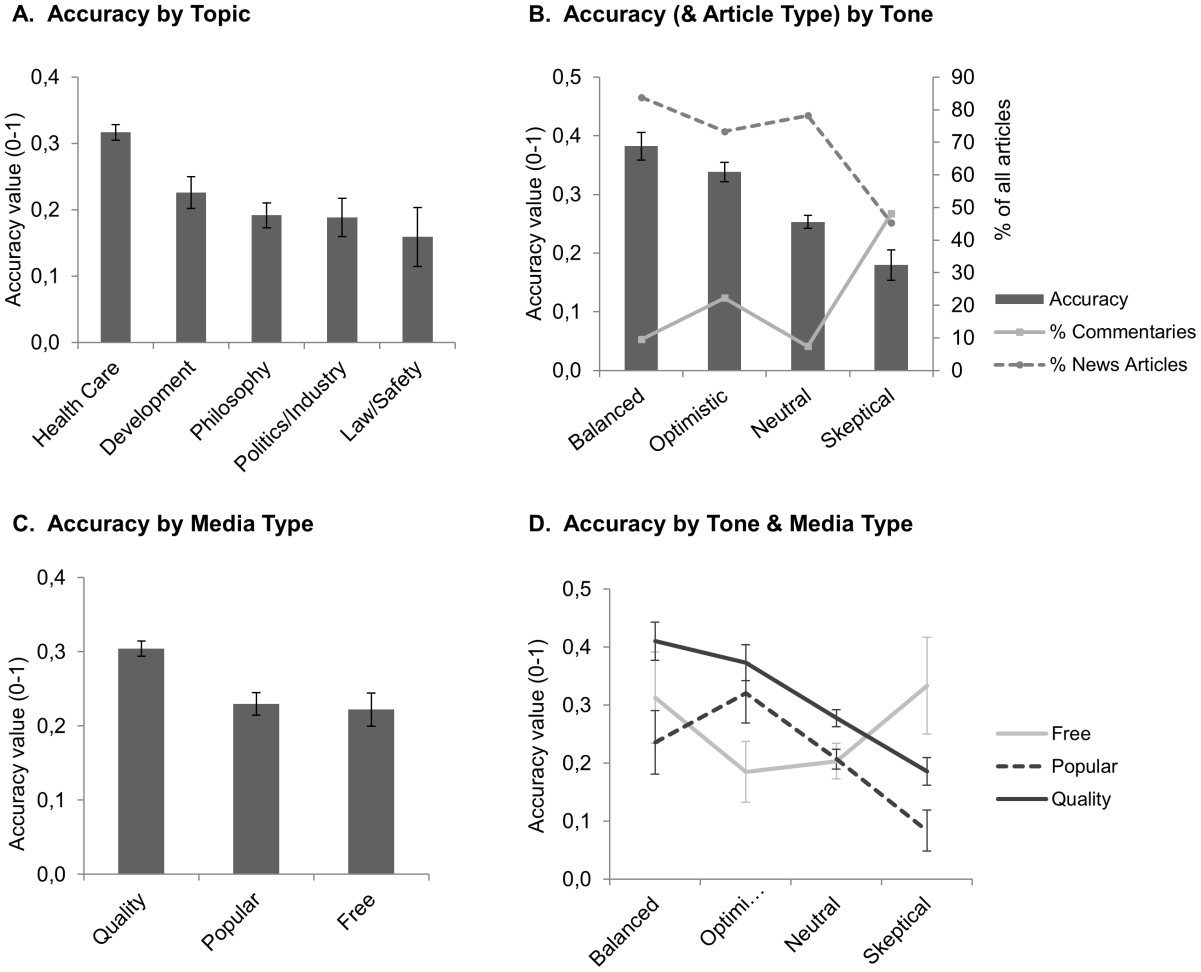
Average accuracy values by Topic, Tone, Media Type and by Tone per Media Type. **A**. The average accuracy values for the different Topics. **B**. The average accuracy values (bars, left vertical axis) for the different Tone categories. The proportion of News articles and Commentaries (as % of all articles) is additionally plotted for each tone category (lines, right vertical axis). **C**. The average accuracy values for the different Media Types. **D**. The average accuracy values by Tone category for the three newspaper types separately. All graphs: error bars indicate s.e.m.

A Kruskal-Wallis test showed that the effect of topic on accuracy was significant (ACCURATE by TOPIC: *H*(4,1070) = 39.3534, p<.001) The mean values for the composite variable ACCURATE (Figure 3A) running from 0 to 1 show that articles reporting on health care issues are most accurate (0.32), followed by development (0.23), philosophy (0.19) and politics/industry (0.19), and is least accurate for law/safety topics (0.16). Behrens-Fisher-Test revealed that the main effect of accuracy is explained by higher accuracy for health care articles compared to all other topics (p<.05 in all four comparisons), all other pairs were not significantly different.

#### 3.2 Critical view

Articles reporting on different topics had significantly different distributions of tone (Figure 2B): TONE by TOPIC, X^2^(12) = 47.0, p<.001. Articles reporting neuroscience research with topics related to learning/development were mostly optimistic (24%) or neutral (64%) and rarely balanced (6%) or skeptical (6%). On the other extreme, articles with topics related to law and safety were rarely optimistic (9%) and most often balanced (21%) or skeptical (18%). Topics about politics/industry or philosophy had the highest proportions of skeptical articles (24% and 23%, respectively).

#### 3.3 Accuracy by Tone

To test the relation between Tone and Accuracy directly, we performed a Kruskal-Wallis test on the ACCURATE values with TONE as independent variable. This analysis revealed a main effect of tone on accuracy: *H*(3,1076) = 37.4788, p<.001. Figure 3B (left y-axis) shows that balanced and optimistic articles are the most accurate (0.38 and 0.34, respectively), followed by neutral articles (0.25), and skeptical articles were the least accurate (0.18). Behrens-Fisher-Test revealed that all pairs differed significantly (p<.05) from each other, except “Optimistic” and “Balanced”.

To gain further insight in the origin of this effect of Tone on Accuracy, we investigated whether a third variable could explain this relation. We expected that different article types, and News articles versus Commentaries in particular (see table 5), would inherently influence Tone and Accuracy, as the aim of news reports is different than for commentaries. Accuracy was significantly different per Article Type *H*(2,1077) = 146.077, p<.001, which was explained by News being significantly more accurate (0.33) than both Commentaries (0.13) and Other articles (0.09). Different Tone categories had significantly different distributions of the different article types: TONE by ARTICLE TYPE, X^2^(6) = 168.9, p<.001. The most notable observation is that skeptical articles are less often News articles (45%), compared to all other Tone categories (Neutral 78%, Optimistic 73%, Balanced 84%, see dotted line and right y-axis in Fig 3B). Together, these findings indicate that the lower accuracy of skeptical articles is probably at least partly due to a lower proportion of the more detailed News articles, and vice versa, the higher accuracy of balanced and optimistic articles is related to a higher proportion of News articles.

### 4. MEDIA TYPE: Is reporting on neuroscience in free and popular newspapers less accurate and critical than in quality newspapers?

#### 4.1 Accuracy

Analysis of Variance showed a main effect of Media Type on accuracy: *H*(2,1077) = 7.72, p<.021. As shown in figure 3C, the mean level of ACCURATE was lower for popular (0.22) and free (0.21) than for quality newspapers (0.30), Behrens-Fisher multiple comparisons of means indicated that quality newspapers differed significantly from free newspapers (p<.05), but not from popular newspapers. Moreover, free newspapers and popular newspapers did not differ from each other.

#### 4.2 Critical view

Articles on neuroscience research had significantly different distributions of tone in the different newspaper types (figure 2C): TONE by MEDIA TYPE, X^2^ (6) = 44.6, p<.001. Free newspapers had a clearly higher proportion of optimistic articles (28%), compared to 16% in quality and 17% in popular newspapers. Quality newspapers had highest proportions of skeptical (16%) and balanced (14%) articles, which were infrequent in popular (5% skeptical, 7% balanced) and free (4% skeptical, 10% balanced) newspapers.

#### 4.3 Accuracy by Tone for the different media types

As described in section 3.3, for all newspapers together, we found an effect of tone on accuracy in the direction of balanced and optimistic articles expressing highest levels of accuracy, and skeptical articles the lowest (Fig 3B). The MEDIA TYPE analyses in the current section (fig 3C and 2C), however, show that quality newspapers are least optimistic but have highest accuracy, and vice versa, free newspapers are most optimistic but less accurate. This suggests that the effect of Tone on Accuracy may not be the same for the different newspaper types, as also appears from the line graphs in figure 3D. We tested this using a 2-factor ANOVA (Tone, Media Type) on ACCURATE values and found main effects for both Tone (*F*(3,1068) = 4.88, p = <.005) and Media Type (*F*(2,1068) = 5.30, p<.005), however the interaction was non-significant (*F*(6,1068) = 0.81, p = .56), so the suspicion of different effects of Tone on Accuracy for the different media types was not backed up by the statistics. Since there is no generally accepted non-parametric test for group interaction effects, a normal two-way ANOVA was used to test this interaction effect. This ANOVA (Tone, Media Type) on ACCURATE values did not find a significant interaction effect (F(6,1068) = 0.81, p = .56). Since a parametric test generally has more statistical power than a non-parametric test, we can confidently conclude that the suspicion of different effects of Tone on Accuracy for the different media types was not backed up by the statistics.

## Discussion

### How accurate and critical are newspaper articles on neuroscience research?

From our results across all 1080 articles the newspaper coverage of neuroscience appears to be not very accurate, and not very critical. Across all articles, only 13% had a balanced tone. In other words, only about one 8th of the articles discussed both benefits as well as limitations of the research. A majority of 57% was neutral in tone, 13% had a skeptical tone, and 17% were optimistic. The low proportions of critical (balanced) articles are very similar to those for the general newspapers in Racine and colleagues [29]; they found 72% to be “uncritical”, which would correspond to our categories “neutral” and “optimistic” combined, which gives 74%. They found slightly more balanced articles: 22% versus our 13%. Also in a larger-scale follow-up study, they found that articles most frequently had an optimistic or neutral tone [30]. Although not including a critical evaluation, neutral articles are informative without biasing a certain interpretation. These results together indicate that UK and Dutch newspaper articles on neuroscience are comparable in overall tone. Racine and colleagues have related this general lack of critique, also emphasized by their findings of very limited discussion of ethical issues, to a conflict of interest between the social demand for research and the value of balanced scientific reporting [46], [47].

On average, the newspaper articles we analyzed had low accuracy (0.27 on a scale from 0–1), which means they included only a very limited amount of research details such as specification and explanation of the used technique (but see below for a discussion of the limitations of the “accuracy” variable used in the current study). Like the limited critical view, the low accuracy is also consistent with previous research [30], [31]. This indicates that in general, newspaper readers do not get informed well about details that would enable them to judge the quality and meaning of the research. A possible consequence is that the public might not be able to distinguish validated knowledge about the brain from myths. A survey of neuroscience literacy showed that the general public is indeed not well informed on neuro-imaging techniques [16]. The same survey also showed that reading newspapers increased correct knowledge about the brain; however, this increase was still rather limited. This indicates that there is much opportunity for improving communication about brain science through daily newspapers, although it should be noted that this survey study is 12 years old and the public's knowledge about the brain may very well have improved since then. In the following, we will further specify our findings in terms of how timing, topic and newspaper type play a role in the limited critical view and low accuracy of neuroscience reporting.

### Is reporting on neuroscience less accurate and critical during news waves than during regular periods?

Our analysis of the critical tone of articles during news waves shows that articles reported during these waves are equally often balanced as in regular reporting periods. We regarded balanced articles as most critical, as they discuss both the possibilities as well as the limitations, and thus best enable the readers to form realistic beliefs and expectations. The media dynamic during heightened media attention therefore does not compromise the amount of balanced articles published. It should be noted however, that during both news waves and regular reporting periods, the proportion of balanced articles is low.

We do find that the tone of articles during news waves is more often optimistic and less often neutral. This increase of optimistic articles at the expense of neutral articles points to increased positive reporting, which was not accompanied by a higher accuracy. This may be concerning as we found that across the total sample of articles, optimistic articles had higher accuracy (see the effect of tone on accuracy in figure 3b, which will be further discussed below). The lower accuracy during news waves strengthens the notion of a positive bias, as the increased optimism may not be grounded in sufficient research details.

In sum, as predicted, our findings show that certain journalistic values are compromised during news waves [33], [34]. The increased optimism without increasing accuracy may indicate that positive information regarding neuroscience is not checked as well during news waves as during regular periods of reporting. The unchanged proportion of balanced articles shows that representing both sides of a story (or a research finding, in this case) is not compromised specifically during news waves, but is very low in general. Although they did not define “hype” as a period in time with increased media reporting, Partridge and colleagues [32] found a similar over-optimistic coverage of neuroscience research that they refer to as a “media hype” related to neuro-enhancement: more positive aspects were mentioned compared to risks or limitations, and the optimism did not seem to be based on solid research evidence. These previous findings, together with the increased optimism found in the current study, suggest that journalists should be cautious during news waves not to be more optimistic than allowed by the facts. Previous research has shown that overly optimistic reporting in some cases starts with the researchers themselves [24], for example by overstating clinical applicability in the conclusions. Therefore, our findings further emphasize that researchers should be extra careful during news waves to convey the right factual basis and tone, to prevent overly optimistic reporting.

### Does accuracy and critical view of articles depend on the reported topic?

The results demonstrate that critical view and accuracy depend on the topic of the article. A notable observation is the high proportion of optimistic articles on brain development and learning, which was as predicted. This may be related to the susceptibility of the educational practice to misconceptions or “neuro-myths” [12]. Dekker and colleagues found that teacher's general knowledge about the brain was predicted by how often they read popular science articles in the media. This indicates that the tone of media articles, which is often optimistic for topics that interest teachers, has the potential to strongly influence a teacher's attitude towards neuroscience findings. This line of thinking is supported by the finding in Dekker et al. [12] that a higher general knowledge about the brain (which was predicted by reading science articles in the media) was related to a higher belief in “neuro-myths” about the neural basis of learning. Therefore, extra care should be taken in communication of topics that interest teachers, to provide balanced information to enable teachers to develop a critical attitude toward “brain-based” teaching methods.

With regard to reporting on neuroscience related to health topics, we found a high proportion of neutral articles, and a relatively high accuracy, which is consistent with findings of Racine et al. [29]. As raised by Borgelt and colleagues [38], inaccurate transfer of neuro-imaging may pose important risks for (mental) health care, such as inappropriate use of brain scans for clinical diagnostics [48]. Our findings of mostly neutral and relatively accurate newspaper coverage of neuroscience related to health care are positive in this regard, as they indicate that health topics are presented in the media relatively accurate (that is, more detailed than other topics). If insights in mental illnesses provided by neuro-imaging techniques are accurately transferred, it may help to reduce stigmatization attached to psychiatric illnesses such as major depression disorder, as it increases the “objectification” of such disorders [38], [39]. However, several other studies have shown effects in opposite directions [18], for example that (neuro)scientific evidence for a mental illness leads to increased community rejection [49], reduced response to treatment [50], and increased individual responsibility for addiction problems [51].

Other notable observations were the low proportion of optimistic articles reporting on topics related to law and safety, and frequent skepticism in articles related to philosophical issues, law, politics and commercial use of neuroscience. These observations are in line with the result of a survey among the UK general public [19] and shows that a general public skepticism on the use of neuroscience in these fields may also be present in the Netherlands. These same topics also expressed the lowest accuracy, especially topics related of law and safety contained only very few research details. To interpret the skeptical and inaccurate reporting of these topics, we will consider two post-hoc findings that provide more insight in the relation of tone, accuracy and topic. One post-hoc finding indicates that especially negative (skeptical) articles tend to be not very detailed. The optimistic and balanced articles, that both include positive aspects of the brain research covered, are significantly more detailed. Optimism in media coverage of neuroscience therefore seems to be warranted more than would be expected, as it is justified by a higher level of detail that provides the scientific basis for the optimistic tone. This conclusion is unexpected as previous research associated optimistic coverage with limited accuracy [32], [35]. However, it should be noted again that during news waves, increased optimism was not accompanied by increased accuracy, indicating that optimism in these periods of heightened media attention may be overly enthusiastic.

The second post-hoc analysis looked at the relation between tone, accuracy and the type of article. As mentioned in the introduction, different article types have different communication goals [41]. News articles aim to inform readers about events, commentaries aim to provide context to interpret developments or events. Moreover, commentaries are more focused on communicating an opinion on an event, rather than the event itself. As predicted, news articles were more accurate than commentaries. We also found a relation of tone and article type: optimistic articles were more often news articles whereas skeptical articles were more often commentaries. Relating this to topic, it seems that the most skeptically covered topics are often discussed in commentaries, which is plausible as law and safety, philosophy and futuristic scenarios, and political and commercial use of neuroscience are all topics that would fit well in commentary articles. Although this may explain the origin of the skeptical and inaccurate reporting, at the same time it indicates that caution is warranted for a negative bias in communicating neuroscience research in relation to these topics, as the lack of accurate articles does not provide the public with enough basis to judge the foundations for this skepticism.

### Is reporting on neuroscience in free and popular newspapers less accurate and critical than in quality newspapers?

As predicted, neuroscience reporting in free and popular newspapers was less accurate compared to quality newspapers. In regards to tone, popular newspapers were more often neutral compared to quality papers, and free newspapers were more often optimistic compared to quality newspapers. Articles in both free and popular newspapers were less often skeptical and balanced. These results suggest that articles in the newspapers that reach most people, the free and popular newspapers, have the lowest accuracy and critical view. It should be noted though that although the quality newspapers have a lower circulation, they report more than half of all articles on neuroscience, which compensates at least partly for the higher circulation of the free and popular newspapers. In other words, readers of quality newspapers are informed more often, more detailed and more critically about neuroscience research; whereas readers of popular and free newspapers, although more in number, are informed less often, less detailed and less critically. It should be noted that the differences in accuracy across newspaper types are *relative* differences, and that also in quality newspapers, average accuracy is rather low. Still, the lower accuracy and lower proportion of balanced articles in free and popular newspapers indicates that scientists should be extra careful about correct translation of their research when interacting with those newspapers, especially considering the high number of people that read these papers.

### Suggestions for future research

With regard to overall accuracy and tone of newspaper articles on neuroscience, the current results from the Dutch media generalize many of the earlier findings from UK and US media [28], [29], [30]. The novel analysis of news waves in the current study can now be applied to study media hype dynamics of neuroscience reporting in other countries as well.

In the introduction, we described the various steps in the translation process at which miscommunications can arise. In future research, it will be important to investigate how these different steps are related, to enable more specific recommendations for improving communication of neuroscience research. For example, related to the first and second steps (limitations of the technique and effect of choices in study design and analysis approach), it will be important to focus follow-up research on specific neuroscience techniques, such functional magnetic resonance imaging (fMRI). In this way, coding the “accuracy” of press articles can include more specific aspects of a certain neuro-imaging technique. In the case of fMRI, as also pointed out by Beck [17], a crucial detail that is important to convey is the choice of the experimental contrast that produced the reported brain activity. The information that such activity is always relative to something else (e.g. another “control” condition) is crucial to interpret the meaning of the brain activity. Other important details to include in future studies focused on fMRI are the number of subjects (as in [30]), and when a clinical group is mentioned in the discussion of the research, whether or not this is justified. Another direction for future research is to focus on specific topics, to enable specifying “accuracy” as meaningful for social issues in a specific context. For example, when focusing on reporting of neuroscience results in the context of law and free will, more specific details can be included to measure accuracy of the communication such as discussion of the legal background, or of how the experimental design represents real-life decision making. As already done for certain specific topics in other studies (e.g. [26]), another important direction for future research is to include the stage of press releases and relate these to both the scientific articles and forthcoming newspaper articles (see also [24], [27], [35], [52], [53]). In a recent study that related newspaper articles to the scientific press release they were based on, showed a high incidence of literal overlap (“copy-paste”), depending on the newspaper [54]. It will be interesting to perform such an analysis specific for neuroscience reporting, and to investigate whether there is a higher incidence of copy-pasting from press releases during news waves.

### Limitations of the present study

Firstly, the accuracy construct used in the present analysis has several limitations. As the current aim was to include all articles reporting on neuroscience, we were limited in which research details we could include. Many details of specific types of experiments were impossible to code for all articles, and as suggested above, should be included in future, more focused research. Secondly, as already mentioned, the communication process of neuroscience to daily life applications is more complex than the chain of steps sketched in the introduction. For example, parallel to the path of scientific journals-press release-media reports, there is also direct translation of research to practice and society, e.g. through experts in clinics or consultants in governmental departments. In addition, the way the public interacts with scientific information is also complex. Green and Clemence [55] analyzed this interaction for a scientific (in their case, genetic) discovery and found that transmission of the discovery was strongly influenced by lay people's pre-existing beliefs and attitudes. A relevant review in this context underscores the impact of pre-existing beliefs, by showing that neuroscience evidence in the context of personhood is integrated with the public's prior understanding of this concept, rather than changing it [18]. In other words, the complex nature of the lay public's interaction with media reports may reduce the impact of media reporting per se, indicating the importance of more future research into media reporting in interaction with the public's beliefs, especially as neuroscience results are open to multiple interpretations.

### Conclusion

It may be unavoidable that results from neuroscience research are generalized and simplified to inform the general public. Therefore, the current challenge is to ensure that the simplified message is still correct [17], or at least, correct enough to avoid generating misconceptions. Empirical research to show the weaknesses of the translation process from the scientific research to the press coverage is important to provide starting points for researchers as well as communication professionals (those responsible for press releases) and (science) journalists to better face the challenge of conveying this simple-but-correct message, although the complex interaction of the public's prior beliefs with the “new” information should also be kept in mind. A general recommendation of the current research to researchers and media professionals is to become more aware of their own role in conveying neuroscience research results accurately and critically to the media. And more specifically, a subset of the coding questions as presented in the current article (table 2, e.g. questions 1–5, question 11, and question 12) could be used as a checklist by these groups to ensure that at least these accuracy and critical tone elements are covered in their press releases or communication messages.

To address the questions we asked in the introduction, the findings of the current media-analysis have provided a basis for the following specific recommendations for science communicators and journalists as well as researchers:

1. *Related to timing: Should one be extra careful during news waves?* Caution is indeed warranted during periods of heightened media attention (news waves), as reporting is more sensitive to positive bias;
2. *Related to the topic: Should accuracy and critical view be guarded more strongly for certain topics compared to others?* Attention should be paid not to follow topic-related biases in optimism (learning, development) or skepticism (law, philosophical issues, commercial use of neuroscience). Covering of neuroscience related to health issues is relatively accurate but could be improved in critical view;
3. *Related to the newspaper type: should scientists be extra alert in guarding correct communication to free and popular newspapers?* Researchers should keep in mind that overall accuracy of reporting is low, and especially articles in popular and free newspapers provide minimal amount of details and balanced views. This indicates that researchers themselves may need to be more active in preventing misconceptions to arise, especially when interacting with more popular media.

In sum, this article provides the necessary information to improve the awareness of researchers, communication professionals, and (science) journalists about the potential pitfalls in the translation process from neuroscience research to media coverage.

## Supporting Information

Appendix S1 **Detailed coding instructions.**
(DOCX)
