# Supplementary material for: Media Reporting of Neuroscience Depends on Timing, Topic and Newspaper Type
Source: PLoS One. 2014 Aug 12;9(8):e104780. doi: 10.1371/journal.pone.0104780 (PMC4130600; doi:10.1371/journal.pone.0104780)
Supplement: Appendix S1 — Detailed coding instructions. (DOCX) [file pone.0104780.s001.docx]

**Appendix S1: Detailed coding instructions**

*Used in media-analysis on neuroscience reporting, van Atteveldt et al.*

**A.** For all articles selected by the search string (see main Materials and Methods), check the following criteria to ensure that only relevant articles are included in the analysis:

***When is an article relevant?***

- Articles on the brain or brain research

- Articles in which brain researchers are cited

***When is an article NOT relevant?***

- Articles in which “brain scans” of publicly known people (e.g. famous people that were in accidents, sportsmen) are the only reason the article was selected by the search string)

- When brain damage or disease is mentioned in a different context than research, e.g. an accident

**N.B.** Mail the researchers in cases of doubt about relevance

**B.** For all the relevant articles, please answer the 14 questions following the guidelines below.

**Coding scheme**

1. ***What is the article type***

These are standard categories for the coders: News report, Background, Person in the news, Editorial comment, Comment by newspaper columnist, External comment, Reader’s letter, Service journalism (book reviews, etc.)

1. ***What is the main topic of the article?***

**Industry/ Politics. Brain research used for commercial purposes or influencing customers/voters, e.g.:**

- - Neuro-marketing (e.g. consumer behavior, consumer choices, neuro-esthetics, commerce)
  - Effect of advertisements in brain (e.g. activation of reward areas, etc)
  - Neuro-economy (decision-making, investing)
  - Job interviews (scans on which employer is supposed to see whether applicant would be qualified)
  - The political brain: how do people choose a politician/party, ratio vs. emotion, etc

**Philosophy/ Futuristic/Nature-Nurture/ Science Fiction.** Articles in which a certain discussion is central (e.g. instead of an application): this can be about ethics, philosophy, nature vs. nurture, or futuristic scenarios, e.g.:

- - Neuro-ethics, bio-ethics (incl. use of test animals)
  - Future scenario’s (cyborgs, brain-computers, superhumans, brain-chips, etc)
  - Free will (if the article’s main focus is philosophical)
  - Discussions with regard to brain and behavior (why am I the way I am?), e.g. biology vs. psychology, determinism vs. free will, etc.

**Health care/ public health**. Health related issues, e.g.:

- - Treatment of psychiatric or neurological disorders (e.g. through psychopharmacology/medication, neuro-feedback, deep brain stimulation, magnetic stimulation)
  - Prevention (e.g.: biomarkers, nutrition)
  - Diagnosis
  - Public health: health education, sexual education, sexual orientation

**Law/ Safety.** Issues on applying neuroscience in law/courtroom and related to safety or security. E.g.:

- - Interrogation, investigation, security (e.g.: lie detection, thought reading, terrorism)
  - Use of brain scans in courtroom: brain scans as evidence, accountability/free will (in case it is focused on crime)
  - Crime, criminal investigation, psychopathy, forensic psychology/psychiatry
- **Learning/ Development**. This includes:
  - Education (applications in the classroom/trainings, e.g. brain-based learning, brain-friendly learning, BrainGym, neuro-education, brain training).
  - Development: baby brain, child’s brain, adolescent’s brain, aging etc
  - Talent, learning skills, IQ etc
  - Parenting and pregnancy (e.g.: pedagogy, caring, neglect)
- **Other:** when none of the above categories is applicable. Mail researchers in this case.

**N.B. It may be possible that multiple categories seem to apply, e.g Law/Safety and Philosophy (e.g. related to free will). Choose the most important issue of the article, e.g. by asking why the research is being discussed**

1. ***On what technique does the article report?***

- **Functional MRI** (fMRI, fMRI-scans) **NB, structural/anatomical MRI belongs to anatomical**

**NB2: if it literally says “brain scan” it should be coded as MRI unspecified.**

- **Brain scan (MRI unspecified):** if no distinction is made between functional and anatomical. E.g. technique described as: MRI (without mentioning of functional or structural/anatomical), brain scans/scanner, brain images, MRI-images, imaging, imaging study/research, neuro-imaging.
- **Anatomical:** structural MRI, anatomical MRI , anatomical scans, DTI, diffusion tensor imaging, diffusion imaging, fiber pathways, brain anatomy post-mortem brain/brain tissue
- **Electro-encephalography**, EEG, brain waves, ERP, brain potentials, electrical waves, electrodes **on** the head
- **Magneto-encephalography**, MEG, magnetic waves, (magnetic) sensors on the head, MEG-scanner
- **Neurostimulation** (not specified)
- **Magnetic stimulation,** TMS, transcranial magnetic stimulation, magnetic pulses, magnetic shocks
- **Electrical stimulation,** deep-brain-stimulation (DBS), electrodes **in** the head, electroshocks, transcranial direct current stimulation (tDCS)
- **Positron-emission tomography**, PET, PET-scanner, radio-active tracers, SPECT
- **Brain-computer-interface** (BCI), brain-machine-interface (BMI), robot, robot-arm, brain-chip, neuro-feedback
- **Psychopharmacology:** the effect on the brain of the presence of, or application of, a certain substance, such as hormones (testosterone, oxytocin, etc), medication/drugs (antidepressants, Ritalin, XTC, etc), nutrients, enzymes, bacteria, protein etc.

- **Other**: if a technique is mentioned, but none of the above. E.g.: single-cell recordings, multi-unit activity, patch-clamp, (computer) simulations, neural network models, optogenetics, NIRS, optical imaging, cooling, neuro-genetics).
- **Unspecified:** if no technique is explicitly mentioned.

**NB If multiple techniques are mentioned/described, code the technique of the research study that is the main topic of the article. In doubt: mail the researchers.**

1. ***Is the technique explained?***

This question specifically concerns the **technique**, not the general research design or explanation of the experiment.

Answer options:

- **Yes** (in 2 or more sentence)
- **Minimal** (1 sentence)
- **No** (not at all)

**N.B.** When other techniques are mentioned/explained in a separate sentence (in addition to the technique used in the reported research) with the purpose of making the central technique more clear, than choose “yes” (2 or more sentences)

1. ***Does the article report on the healthy brain or a brain disorder?***

**- Healthy-general:** healthy adults were studied, and/or an article does not mention any relation to a disorder.

**- Healthy-development:** when a certain age Group is studied but unrelated to a disorder, E.g. healthy fetus or normal aging.

**- Disorder-psychiatric:** any disorder you would see a psychologist or psychiatrist for. These disorders are classified in the DSM manual. Examples: schizophrenia, psychosis, hallucinations, delusions, autism, Asperger, ADHD, depression, bipolar disorder, addiction, eating disorder (obesities, anorexia, bulimia), posttraumatic stress disorder, stress, anxiety disorder, phobia, burn-out, Alzheimer/dementia, insomnia, aggression, psychopathy, personality disorder, borderline, anti-social disorder, obsession, obsessive-compulsive disorder (OCD).

**- Disorder-general**: any disorder you don’t need a psychiatrist/psychological care, but rather a neurologist or a remedial pedagogue. Examples: Parkinson, MS, tumor, stroke, TIA, hemorrhage, , locked-in syndrome, blind, deaf, dyslexia, dyscalculia, aphasia, stuttering, epilepsy, narcolepsy, synesthesia, cognitive impairment (e.g. related to Down syndrome, or fragile X-syndrome), learning disorder, gifted, phantom pain, chronic pain, migraine, tinnitus, coma, vegetative state, spina bifida, hydrocephalus, meningitis, lesion.

- **Disorder: both psychiatric and general**: in case both are mentioned.

**N.B.** In case both the healthy brain as well as a disorder is mentioned, choose a “disorder” category (the healthy brain is probably for comparison/control purposes). The same applies to cases when both the healthy (adult) brain and the developing/aging brain is mentioned, choose “development” in this case (the adult brain is probably used for comparison).

It can occur that a disorder is mentioned, but strictly speaking the research has been performed in healthy adults only. In this case, choose a “disorder” category: as the disorder is probably the application or purpose of mentioning the brain research.

**N.B.** The disorder/age group has to be mentioned explicitly when choosing for a disorder/development category.

1. ***What change or effect was found by the research?***

This question can apply both to changes/effects in the brain as well as in symptoms/functions/behavior. It can also apply to prospects/expectations of changes or effects. It may apply to effects within an individual/group (e.g. treatment related) or between groups (e.g. Higher/lower brain activity in a certain group compared to a control group).

**N.B.** It’s not always the case that a study is reported that intended to find a certain effect, in that case choose “no effect intended”.

**- Improvement/increase**. N.B. “increase” can also be negative, e.g. increase in symptoms = worsening (in those cases, the effect on the (test) person is most important: is he/she doing better = improvement, doing worse: worsening). Better prevention/prediction of a disorder or disease also falls in the category “improvement”.

**- No change or effect.**

**- Worsening/decrease.**

**- No effect intended** (when the other options are not applicable)

**N.B.** Mail researchers in case of doubt, it can occur that improvement as well as worsening are mentioned.

1. ***What brain function was investigated?***

- **Memory (**e.g. healthy: recollection, working memory, short-term memory, long-term memory. e.g. disorder: dementia, Alzheimer )
- **Motor functions** (e.g. healthy: moving, rhythm, dancing, walking, grasping, sports, gestures. e.g. disorder: Parkinson, MS, paralysis, locked-in)
- **Attention** (e.g. healthy: concentration, being alert, focusing, distracted, e.g. disorder: ADHD)
- **Sleep/consciousness** (general: different states of consciousness, e.g. sleep, coma, awake/alert, meditation, etc. e.g. healthy: REM sleep, deep sleep, sleep oscillations, sleep cycle, dreaming. E.g. disorder: insomnia, vegetative state (minimal consciousness), coma, chronic fatigue, epilepsy, fainting)
- **Perception**/**Illusions** (e.g. healthy: seeing, hearing, smelling, feeling, tasting, e.g. disorder: pain, migraine, blind, deaf, tinnitus, hallucinations, delusions)
- **Social/Emotions** (e.g. healthy: empathy, imitation, mirror neurons, emotion, mood, stress, friendship, love, sexual orientation, gender issues, sex, guilt, lying, personality, identity, character, moral behavior, e.g. disorder: autism, Asperger, PDD-NOS, psychopathy, social anxiety, mood disorder/depression, stress disorder, personality disorders)
- **Cognition** (e.g.: language, reading, learning, writing, speaking, language development, calculating, thinking, knowing, intelligence, IQ, e.g. disorder: learning disorder, mental retardation, highly gifted, dyslexia, dyscalculia, stuttering)
- **Planning**/**Control/Free will.** (e.g. healthy: accountability, inhibition, decision-making, self-regulation, goal setting, impulsivity, planning ahead, religion, free will. E.g. disorder: addiction, OCD, aggression, not accountable, crime)
- **General/multiple** (no specific function is mentioned, but e.g. “workings of the brain” in general. Psychosomatic symptoms also fall under this category, except when the symptoms are clearly related to 1 specific function.

**N.B.** In case multiple functions are mentioned: when there are many, and the article does not focus on 1 function in particular, choose “General/Multiple”. When one function is clearly the central issue and other functions are only mentioned very briefly, cheese the central function. In case of doubt, mail the researchers.

***8. What was the overall tone of the article?***

This question is about whether or not **benefits/limitations of the technique/method** are mentioned or discussed (positive results/effects are coded in question 6). In addition, the tone is defined by the description of how the results can be used: e.g. whether amazing new insights are provided that will solve important problems (optimistic), or mentioning that more research is needed, e.g. in humans (critical). It does not matter who makes the optimistic/skeptical statements (researchers themselves, independent expert, journalist), as long as they are in the article, they contribute to the overall tone.

- **Optimistic**: benefits are mentioned, risks/challenges/limitations are not. Keywords that point to optimistic tone: breakthrough, promising, convincing, robust/solid/strong effects/results, proud, fascinating, recovery, disclosure, exceptional, innovative, novel, pioneering, answers, evidence/proof, clear-cut, obvious, evident, new insight, for the first time, new/better treatment.

- **Neutral**: benefits, risks or challenges are not mentioned.
- **Critical/skeptical**: risks, challenges and/or limitations are mentioned, benefits are not. Keywords that point to critical/skeptical tone: but, risk, skepticism, critique, challenge, reliability, false positives, over-diagnosis, limitation, follow-up research needed, replications needed (e.g. in humans, in larger samples), unclear, subtle, supposed, putative, assumption, future scenario, stigmatizing, stereotyping, brainwashing, neuro-myth, myth, neuro-hype, brain hype, neurophobia, neuromania, brain porn, neurobiologification, adverse effects, side effects, question marks, disappointing, overestimated.
- **Balanced**: both the benefits and the challenges are mentioned. Also choose this option if the benefits of one technique are compared to risks/limitations of another technique.

***9. What species was tested in the research?***

Sometimes, an experiment has been conducted on mice, rats or other test animals, but the results are extrapolated to humans. In questions 9 and 10, we assess whether this is the case in the articles. In question 9, the question is on what species the **scientific** research has been conducted:

***-* Humans**

**- Animals**

**- Not mentioned**

**N.B.,** sometimes, multiple scientific experiments are mentioned that were conducted with different species. Choose “Animal” when animals were tested in at least 1 experiment, while the article reports on the human brain.

***10. Does the article generalize from animal research to human implications? If yes, with or without explanation?***

**N.B. The yes/no answers only apply to articles for which question 9 was answered with “Animals”, please choose “not applicable” in case the answer to question 9 was “human” or “not mentioned!**

In case the answer to question 9 is “Animal”, the following answer options are available:

**- Yes, without explanation**: the article does not mention/discuss the limitations of the animal-to-human generalization

**- Yes, with explanation:** the article mentions/discusses the limitations of the generalization

**- No:** Experiments on humans and animals is strictly separated in the article.

***11. What is the main message of the article?***

This question concerns the **most important** message of the articles. It may occur that more than one of the answer options applies to a certain extent; in this case extract the most important message.

***- Emphasizing group differences***. This applies when either two groups are compared, or the articles talks about the “xx brain”, where xx can be something like obese, female, addictive, etc., and the message is that this “xx brain” works differently than the “normal” brain.

Examples: gender/orientation/age group (homo/hetero/pedophile, female/male, adolescents/elderly), addictions (alcoholics, smokers), behavior (criminals, aggressive, impulsive), disorders (autistic, schizophrenic, psychopath, ADHD).

***- (New) application of brain research.*** E.g.: new teaching method, lie detector, new treatment or therapy. It can also be a recommendation/advice about an application.

***- Effect of a substance on the brain.*** Examples: drug/medication (XTC, Ritalin, Prozac, aspirin, etc), nutrition and supplements (fast food, omega-3 fatty acids, chocolate, caffeine, alcohol, anti-oxidants, etc), hormones (oxytocin, cortisol, testosterone, progesterone, etc)

**N.B.** Substances can be mentioned in the context of development of a new medication/treatment. If this new application is the most important message, choose “(New) application of brain research”.

***- Rhetoric:*** Brain research is being used to support on argument, e.g. to support a certain policy proposal (e.g. to support music education in schools) or a daily phenomenon, or to explain another effect or research result in a different field.

***-*** ***Other:*** If none of the above applies. Mail the researchers if this occurs.

**N.B.** It can occur that the main message is rhetoric but that other messages are also present. Again, think thoroughly about what is the **most important message.** For example, an article can mention differences in brain development between boys and girls (= group differences), but for the purpose of supporting education policy of separating the sexes for certain lessons. In this case, the **most important** message is **rhetoric**.

**N.B.2** Articles that most importantly want to give an opinion on brain research also belong to this category. This can be either optimistic or skeptical.

**N.B.3. If you choose rhetoric, go back and think again about the Tone (question 8):** when brain research is used as argument in favor of something, and no limitations or disadvantages of the technique/research are mentioned, this is an indication of Optimistic tone (or Balanced when the disadvantages are mentioned).

***12, 13, 14. Which sources are mentioned?***

These questions can be answered with “yes” or “no”. Only choose “yes” if the group in question is actually cited. E.g. in case of “researchers”, if they are literally cited or accurately paraphrased. Mail in case of doubt, especially when it concerns paraphrasing. Also, the cited statements should be about the brain or brain research. E.g. , when people from a certain practice talk about very different topics, this is not relevant here.

**- 12. Are the researchers of the reported work consulted as a source?** Scientists, neuroscientists, brain researchers, neuro-imagers, authors (of the scientific publication in question). Different titles can be used, but they should be the people who conducted the reported research. Other scientists belong to question 13.

**N.B.** In the case of an article that does not report a specific research but on a more general aspect of the brain/brain research, the answer to this question will typically be “no”.

**N.B.2** In case the article reports on a book about the brain/brain research, and the author has not been interviewed, but the article cites from the book, this also counts as “yes”.

**- 13. Are independent experts or someone from the practice consulted as source?** This concerns not the researchers that conducted the reported research, but they can still be brain scientists. E.g., a Dutch professor comments on an American research. Persons from the practice are the ones that will be applying the research findings (possibly in the (far) future). E.g. teachers, parents, trainers, pedagogues, medical doctors, neurologists, neurosurgeons, revalidation-therapists, interrogators, lawyers, judges, police, customs, employers, companies, market researchers, advertisers, policy makers, etc.

**- 14. Is the scientific journal in which the research is published mentioned?** The scientific journal such as Science, Neuron, Neurology, PNAS, Current Biology, Brain, etc.
